# COVID-19 Control by Computer Vision Approaches: A Survey

**DOI:** 10.1109/ACCESS.2020.3027685

**Published:** 2020-09-29

**Authors:** Anwaar Ulhaq, Jannis Born, Asim Khan, Douglas Pinto Sampaio Gomes, Subrata Chakraborty, Manoranjan Paul

**Affiliations:** 1 School of Computing and MathematicsCharles Sturt University 1109 Port Macquarie NSW 2795 Australia; 2 Department for Biosystems Science and EngineeringETH Zurich 4058 Basel Switzerland; 3 College of Engineering and ScienceVictoria University 497603 Melbourne VIC 3011 Australia; 4 Faculty of Engineering and Information TechnologyUniversity of Technology Sydney 1994 Sydney NSW 2007 Australia

**Keywords:** Artificial intelligence, COVID-19, computer vision, review, survey

## Abstract

The COVID-19 pandemic has triggered an urgent call to contribute to the fight against an immense threat to the human population. Computer Vision, as a subfield of artificial intelligence, has enjoyed recent success in solving various complex problems in health care and has the potential to contribute to the fight of controlling COVID-19. In response to this call, computer vision researchers are putting their knowledge base at test to devise effective ways to counter COVID-19 challenge and serve the global community. New contributions are being shared with every passing day. It motivated us to review the recent work, collect information about available research resources, and an indication of future research directions. We want to make it possible for computer vision researchers to find existing and future research directions. This survey article presents a preliminary review of the literature on research community efforts against COVID-19 pandemic.

## I. Introduction

COVID-19, known as an infectious disease is caused by severe acute respiratory syndrome (SARS-CoV-2) [1] and named coronavirus due to its visual appearance (under an electron microscope) to solar corona (similar to a crown) [2]. The fight against COVID-19 has motivated researchers worldwide to explore, understand, and devise new diagnostic and treatment techniques to culminate this threat to our generation. In this article, we discuss how the computer vision community is fighting with this menace by proposing new types of approaches, improving efficiency, and speed of the existing efforts.

**FIGURE 1.**
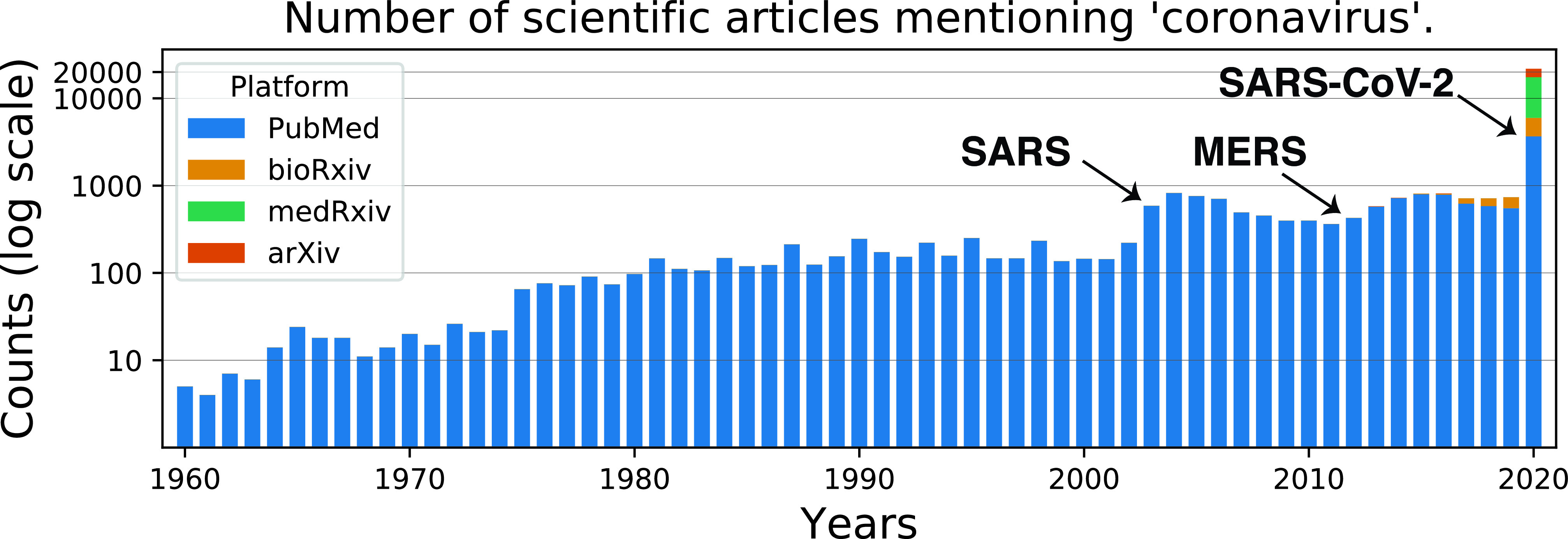
A portrayal of current increase in research articles about coronavirus related research. Since their discovery in the early 1960s, coronavirus research has increased substantially; especially after the SARS outbreak in 2002 made clear their pandemic potential. Previously, the most productive full year was 2004 with 822 coronavirus papers. The SARS-CoV-2 pandemic has caused a leap, with 21,806 articles only in the first half of 2020 (reference date for the analysis was 30 June 2020). Note that the y-axis is displayed in log-scale for visual clarity and that the height of the coloured bars shows their relative contribution.

The scientific response to combat COVID-19 has been far quicker and widespread. A keyword search on PubMed and the major open-access preprint repositories (arXiv, bioRxiv and medRxiv) revealed that in 2019, 735 published papers included the word “coronavirus”. FIGURE 1 illustrates our findings. During the first half of 2020, this number has increased a thirty-fold and rose to astounding 21,806 articles. For comparison, the SARS pandemic, with less than 10,000 confirmed infections and <1,000 deaths, led roughly to a four-fold increase over two years (2002: 221 and 2004: 822). After the occurrence of MERS in 2012 (less than 3,000 confirmed infections and 1,000 deaths to date) a doubling in coronavirus related papers over four years (2011 to 2015) was observed.

The Economist has dubbed the current Herculean task science of the times with the hope that such efforts would help speed up the development of a COVID-19 vaccine [3].

Numerous approaches in computer vision have been proposed so far, dealing with different aspects of combat the COVID-19 pandemic. These approaches vary in terms of their approach to the fundamental questions:

• How can medical imaging facilitate faster and reliable diagnosis of COVID-19?
• Which image features correctly classify conditions as Bacterial, Viral, COVID-19, and Pneumonia?
• What can we learn from imaging data acquired from disease survivors to screen critical and non-critical ill patients?
• How can computer vision be used to enforce social distancing and early screening of infected people?
• How can 3D computer vision help to maintain healthcare equipment supply and guide the development of a COVID-19 vaccine?

The answers to these questions are being explored, and preliminary work has been done.

The contribution of this review article is as follows: This review article classifies COVID-related computer vision methods into broad categories and provides salient descriptions of representative methods in each group. We aspire to give readers the ability to understand the baseline efforts and kickstart their work where others have left. Furthermore, we aim to highlight new trends and innovative ideas to build a more robust and well-planned strategy during this war of our times.

Our survey will also include research articles in pre-print format due to the time urgency imposed by this disease. However, one limitation of this review is the inclusion of the risk of lower quality and work without due validation. Many of the works have not been put into the clinical trial as it is time-consuming. Nevertheless, our intention here is to share ideas from a single platform while highlighting the computer vision community efforts. We hope that our reader is aware of these contemporary challenges. This article is an extended and revised version of the earlier preprint survey [4]. We follow a top-down approach to describe the research problems that require urgent attention. We start with disease diagnosis, discuss disease prevention and control, followed by treatment-related computer vision research work.

We have organised the paper as follow: Section 2 describes the overall taxonomy of computer vision research areas by classifying these efforts into three classes. Section 3 provides a detailed description of each research area, relevant papers, and a brief description of representative work. Section 4 describes available resources, including research datasets, their links, deep learning models, and codes. Section 5 provides the discussion and future work directions followed by concluding remarks and references.

## II. Historical Development

The novel coronavirus SARS-CoV-2 is the seventh member of the Corona viridae family of viruses which are enveloped, non-segmented, positive-sense RNA viruses [5]. The mortality rate of COVID-19 is less than that of the severe acute respiratory syndrome (SARS) and Middle East respiratory syndrome (MERS) coronavirus diseases (10% for SARS-CoV and 37% for MERS-CoV). However, it is highly infectious, and the number of cases is on continuous rise [6].

The disease outbreak first reported in Wuhan, the Hubei province of China, after several cases of pneumonia with unknown causes were reported on 31 December 2019. A novel coronavirus was discovered as the causative organism through in-depth sequencing analysis of samples of patient’s respiratory tract at Chinese facilities on 7 January 2020 [6]. The outbreak was announced as a Public Health Emergency of International Concern on 30 January 2020. On 11 February 2020, the World Health Organization (WHO) announced a name for the new coronavirus disease: COVID-19. It was officially being considered pandemic after the 11 March announcement by WHO [7].

## III. Taxonomy of Key Areas of Research

**FIGURE 2.**
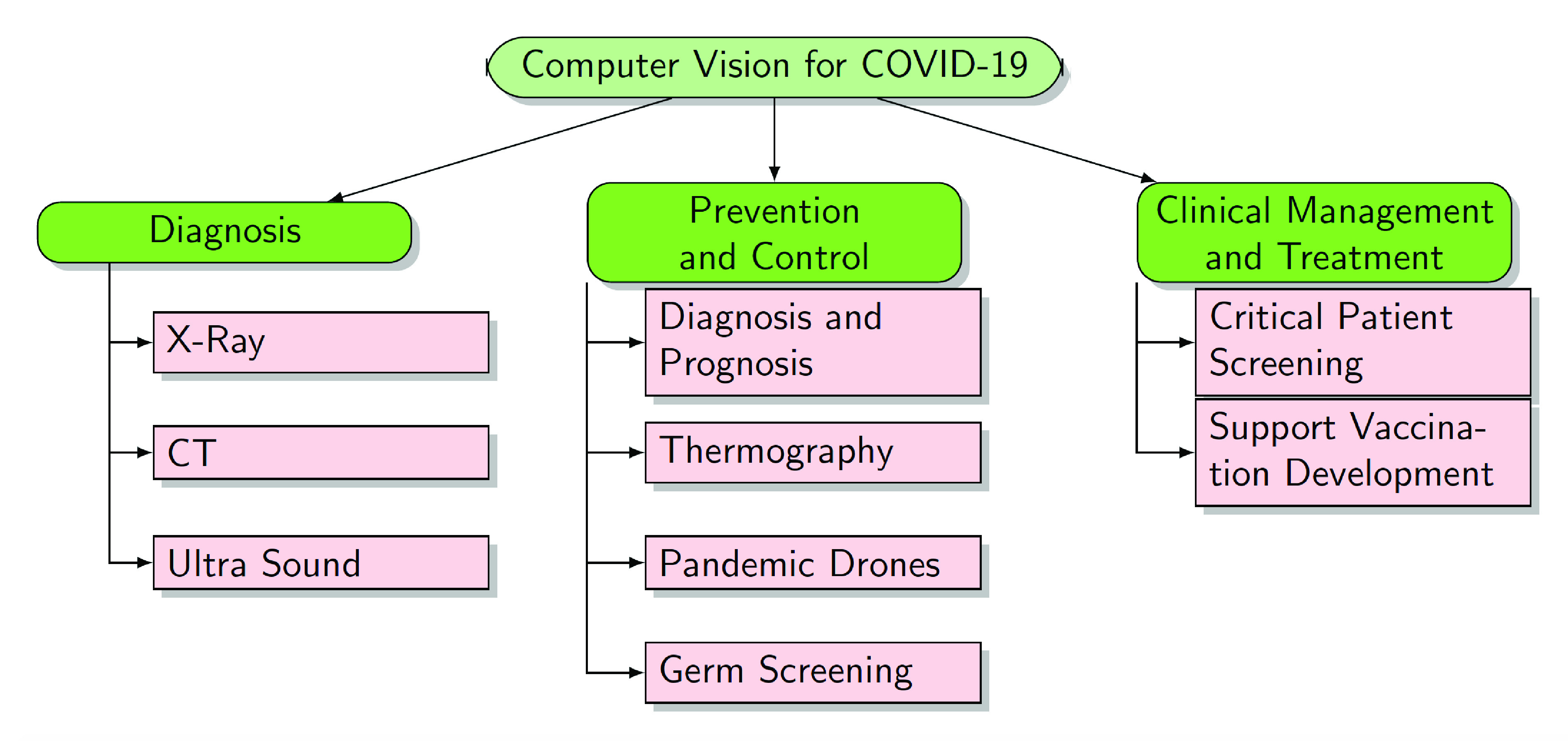
Classification of computer vision approaches for COVID-19 Control. Our survey classifies COVID-19 related computer vision methods into three broad categories.

In this section, we describe the classification of computer vision techniques that try to counter the menace of COVID-19. For better comprehensibility, we have classified them into three key areas of research: (i) diagnosis and prognosis, (ii) disease prevention and control, and (iii) disease treatment and management. FIGURE 2 shows this taxonomy. In the following subsections, we discuss the research fields, the relevant papers, and present a brief representative description of related works.

### A. Diagnosis and Prognosis

An essential step in this fight is the reliable, faster, and affordable diagnostic process that can be readily accessible and available to the global community. According to Cambridge dictionary [8], diagnosis is: “the making of a judgment about the exact character of a disease or other problem, especially after an examination, or such a judgment” and prognosis is “a doctor’s judgment of the likely or expected development of a disease or of the chances of getting better”.

Currently, Reverse transcriptase quantitative polymerase chain reaction (RT-qPCR) tests are considered as the gold standard for diagnosing COVID-19 [9]. During such a test, small amounts of viral RNA are extracted from a nasal swab, amplified, quantified. Virus detection is then performed using a fluorescent dye. Although accurate, the test is time-consuming, manual and requires biomolecular testing facilities which limits its availability in large scales and third-world countries. Care has to be taken in interpreting negative test results. A meta-study estimated the sensitivity over the disease process and found a maximal sensitivity of 80%, eight days after infection [10]. Some studies have also shown false-positive PCR testing [11].

#### 1) Computed Tomography (CT) Scan

An alternative approach is the use of a radiology examination that uses computed tomography (CT) imaging [12]. A chest CT scan is a non-invasive test conducted to obtain a precise image of a patient’s chest. It uses an enhanced form of X-Ray technology, providing more detailed images of the chest than a standard X-Ray. It produces images that include bones, fats, muscles, and organs, giving physicians a better view, which is crucial when making accurate diagnoses.

A Chest CT scan is of two types: namely high-resolution and spiral chest CT scan [13]. The high-resolution chest CT scan provides more than a slice (or image) in a single rotation of the X-Ray tube. The spiral chest CT scan application involves a table that continuously moves through a tunnel-like hole while the X-Ray tube follows a spiral path. The advantage of the spiral CT is that it is capable of producing a three-dimensional image of the lungs.

**FIGURE 3.**
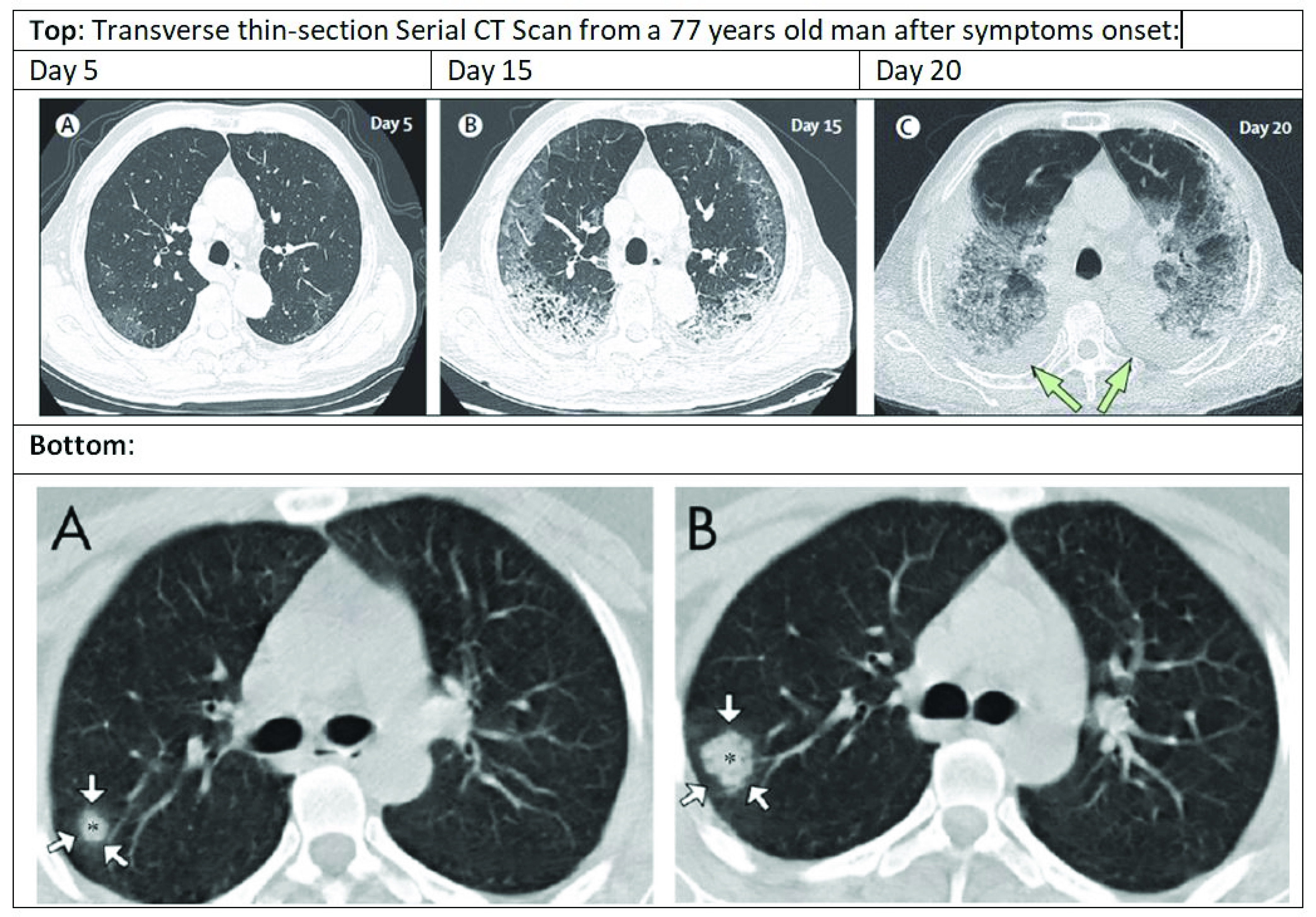
CT images adapted from [6], [18] portray CT features related to COVID-19. Ground glass opacities (top) and ground glass halo (bottom).

Important CT features include ground-glass opacity, consolidation, reticulation/thickened interlobular septa, nodules, and lesion distribution (left, right or bilateral lungs) [14]–[17]. The most observable CT features discovered in COVID-19 pneumonia include bilateral and sub pleural areas of ground-glass opacification, consolidation affecting the lower lobes. Within the intermediate stage (4-14 days from symptom onset), crazy-paving pattern and possibly observable Halo sign become important features as well [6], [11], [12], [12], [14]–[18]. One case of CT images is shown in FIGURE 3 that illustrates ground glass opacities and ground halo features. As the identification of disease features is time-consuming, even for expert radiologists, computer vision can help by automating such a process.

#### 2) Representative Work, Evaluation and Discussion

To date, various CT-scanning automated approaches have been proposed [8], [12]–[16], [18]–[27]. To discuss the approach and performance of the computer vision CT-based disease diagnosis, we have selected some recent representative works that provide an overview of their effectiveness. It is worth noting that they have been presenting different performance metrics and using a diverse number of images and datasets. These practices make their comparison very challenging. Some of the metrics include Accuracy, Specificity, Sensitivity, Positive predictive value (PPV), Negative predictive value (NPV), Area Under Curve (AUC), and F1 score. A quick elucidation on their definition can be useful. The accuracy of a method finds how correct the values are predicted. The precision finds the reproducibility of the measurement; Recall presents how many of the correct results are discovered while F1-score uses a combination of precision and recall for a balanced average result.

The first class of work discussed here approaches diagnosis as a segmentation problem. Chen *et al.* [22] has proposed a CT image dataset of 46,096 images of both healthy and infected patients, labelled by expert radiologists. It was collected from 106 patients admitted with 51 confirmed COVID-19 pneumonia and 55 control patients. The work used deep learning models for segmentation only so that it could identify the infected area in CT images between healthy and infected patients. It was based on UNet++ semantic segmentation model [23], used to extract valid areas in the images. It used 289 randomly selected CT images and tested it on other 600 randomly selected CT images. The model achieved a per-patient sensitivity of 100%, specificity of 93.55%, the accuracy of 95.24%, PPV (positive prediction value) of 84.62%, and NPV (negative prediction value) of 100%. In the retrospective dataset, it resulted in a per-image sensitivity of 94.34%, the specificity of 99.16%, the accuracy of 98.85%, PPV of 88.37%, and NPV of 99.61%. The trained model from this study was deployed at the Renmin Hospital of Wuhan University (Wuhan, Hubei province, China) to accelerate the diagnosis of new COVID-19 cases. It was also open-sourced on the Internet to enable a rapid review of new cases in other locations. A cloud-based open-access artificial intelligence platform was constructed to provide support for detecting COVID-19 pneumonia worldwide. For this purpose, a website has been made available to provide free access to the present model at (http://121.40.75.149/znyx-ncov/index). Table 1 presents a description of the representative techniques for CT based COVID-19 diagnosis.

**TABLE 1:** Representative Works for CT Based COVID-19 Diagnosis

Study	Classification Model and availability	Segnentation Model	Dataset	No of Participants	Deployed	Performance
Jun Chen et al. [22]	(http://121.40.75.149/znyx-ncov/index)	UNet++ to extract valid areas in CT images using 289 randomly selected CT images	46,096 CT images	106 patients with 51 confirmed COVID-19 pneumonia	Renmin Hospital of Wuhan University (Wuhan, Hubei province, China)	Sensitivity of 100%, specificity of 93.55%, accuracy of 95.24%.
Shual Wang et al. [28]	Modified inception [29] with transfer learning available at: https://ainsce-tj.cn/thai/deploy/public/pneumonia_ct.	–	453 CT images of pathogenconfirmed COVID-19	99 patients from Xi’an Jiaotong University First Affiliated Hospital, Nanchang University First Hospital and Xi’An No.8 Hospital of Xi’An Medical College	–	The internal validation achieved accuracy of 82.9% specificity of 80.5% sensitivity of 84%. The external testing dataset showed accuracy of 73.1% with specificity of 67%.
Xiaowei Xu al. [30]	Combination of Two CNN three-dimensional classification models ResNet-18 network [31] +location attention oriented model).	VNET based segmentation model [32]	A total of 618 CT samples were collected: 19, 224 CT samples Flue 1175 CT samples from healthy people	219 from 110 patients with COVID-224 patients with Influenza-A viral pneumonia	–	Model accuracy 86.7%
Ying Song et al. [33]	Details Relation Extraction neural networkDRE-Net + ResNet50 [34] with Feature Pyramid Network (FPN)+ Attention module An online server is available for online diagnoses with CT images by http://biomed.nscegz.cn/server/Ncov2019.	–	777 CT images	88 patients diagnosed with the COVID19101 patients infected with bacteria pneumonia, and 86 healthy persons	–	AUC of 0.99 and recall (sensitivity) of 0.93. Accuracy of 0.86 and F-Score 0.87
Ophir Gozes e t al. [35]	2D deep convolutional neural network architecture based on Resnet-50 [34]	U-net architecture for image segmentation [36]	–	56 patients with confirmed COVID-19 diagnosis	–	0.996 AUC (95% CI: 0.989-1.00) on Chinese control and infected patients. 98.2% sensitivity, 92.2% specificity.
Fei Shan [37]	–	VB-Net” network to segment COVID-19 infection regions in CT scans	249 CI images	249 COVID-19 patients, and validated using new COVID-19 patients	–	Dice similarity coefficients of 91.6% t 10.0% between automatic and manual segmentations
Cheng Jin et al. [17]	Model is available at: www.github.com/ChenWWWeixiang/diagnosis_covid19.	2D CNN based AI system, model name is not specified	970 CT volumes	496 patients with confirmed COVID-19	–	accuracy 94.98% area under the receiver operating characteristic curve (AUC) of 97.91%
Mucahid Barstuganl et al. [38]	Grey Level Co-occurrence Matrix Local Directional Pattern Grey Level Run Length Matrix Grey Level Size Zone Matrix Discrete Wavelet Transform + SVM	–	150 CT images.	–	–	99.68% classification accuracy
Lin Li [24]	(COVNet), was developed to extract visual features from volumetric chest CT RESNET50. Model is available at: https://github.com/bkong999/COVNet	U-Net for segmentation	4356 chest CT images	The datasets were collected from 6 hospitals and 3,322 patients.		The sensitivity and specificity for COVID- 19 are 90% and 96% respectively.
Chuansheng Zheng [39]	3D deep convolutional neural Network to Detect COVID-19 (DeCoVNet) from CT volumes. The developed deep learning software is available at https://github.com/sydneyOzq/covid-19-detection.	Segmented using a pre-trained UNet		UnionHospital,Tongji Medical College, Huazhong University of Science and Technology). Finally, 540 patients		Obtained 0.959 ROC AUC and 0.976 PR AUC.
Shuo Jin [40]	Transfer learning on ResNet-50	segmentation model as 3D U-Net++,		Using 1,136 training cases (723 positives for COVID- 19) from five hospitals	Deployed the system in 16 hospitals in China	AUC 0.991 sensitivity of 0.974 and specificity of 0.922.
Mei et al. [27]	Slice selection CNN with Inception-ResNet-v2 backbone. Followed by disease diagnosis CNN with ResNet-18 backbone. Code for the model: https://github.com/howchihlee/COVID 19_CT		Unpublished	905 patients, from which 419 were COVID positive. 279 patients were used for testing		AUC 0.92, sen-sitivity of 0.84 and specificity of 0.83.

The second type of work considered COVID-19 as a binary classification problem. Li *et al.* [24] proposed (COVNet), to extract visual features from volumetric chest CT using transfer learning on the RESNET50. Lung segmentation was performed as a pre-processing task using the U-Net model. It used 4356 chest CT exams from 3,322 patients from the dataset collected from 6 hospitals between August 2016 and February 2020. The sensitivity and specificity for COVID-19 are 90% (114 of 127; p-value<0.001) with 95% confidence interval (CI) of [95% CI: 83%, 94%] and 96% (294 of 307; p-value<0.001) with [95% CI: 93%, 98%], respectively. The model was also made available online for public use at https://github.com/bkong999/COVNet.

The diagnosis problem was also approached as a 3-category classification task: distinguishing healthy patients from those with other types of pneumonia and those with COVID-19. Li *et al.* [24] used data from 88 patients diagnosed with the COVID-19, 101 patients infected with bacteria pneumonia, and 86 healthy individuals. It proposed the DRE-Net (Relation Extraction neural network) based on ResNet50, on which the Feature Pyramid Network (FPN) [25] and the Attention module was integrated to represent more fine-grained aspects of the images. An online server is available for online diagnoses with CT images at http://biomed.nsccgz.cn/server/Ncov2019.

A recent landmark study was published by Mei *et al.* [27] in *Nature Medicine*. In a cohort of 906 RT-PCR tested patients (419 COVID-positive), a two-stage CNN was combined with an MLP on clinical features (age, sex, exposure history, symptoms) and the diagnostic performance was compared to senior radiologists. A “slice selection CNN” was used to select abnormal CT scans which were subsequently classified by the “disease diagnosis CNN”. Interestingly, fusing a 512-dimensional vector of the CT scans with clinical features yielded a joint model that significantly outperformed the CNN-only model in ROC-AUC and specificity. On a test set of 279 patients, the joint model surpassed senior radiologists in ROC-AUC (0.92 vs. 0.84), while showing worse specificity (83% vs. 94%) and statistically insignificant better sensitivity (84% vs. 75%). The model also correctly identified 68% of positive patients who exhibited normal CT scans according to the radiologists. It hints toward the potential of deep learning to pick-up complex, disease-relevant patterns that may stay indiscernible for radiologists.

Due to limited time available for annotations and labelling, weakly-supervised deep learning-based approaches have also been developed using 3D CT volumes to detect COVID-19. Zheng *et al.* [26] proposed 3D deep convolutional neural Network (DeCoVNet) to Detect COVID-19 from CT volumes. The weakly supervised deep learning model could accurately predict the COVID-19 infectious probability in chest CT volumes without the need for annotating the lesions for training. The CT images were segmented using a pre-trained UNet. It used 499 CT volumes for training, collected from 13 December 2019 to 23 January 2020, and 131 CT volumes for testing, collected from 24 January 2020 to 6 February 2020. The authors chose a probability threshold of 0.5 to classify COVID- positive and COVID- negative cases. The algorithm obtained an accuracy of 0.901, a positive predictive value of 0.840, and a high negative predictive value of 0.982. The developed deep learning model is available at https://github.com/sydney0zq/covid-19-detection.

#### 3) X-Ray Imagery

One drawback of using CT imaging is the need for high patient dose and enhanced cost [43]. The low availability imposes Additional challenges for CT in remote areas and the need of patient relocation and exhaustive disinfection of the scanner rooms (several hours per day) that risk contagion for staff and other patients [44]. These disadvantages call into play chest X-Ray radiography (CXR) as a preferred first-line imaging modality with lower cost and a wider availability for detecting chest pathology. Digital X-Ray imagery computer-aided diagnosis is used for different diseases, including osteoporosis [45], cancer [46] and cardiac disease [39]. However, as it is really hard to distinguish soft tissue with a poor contrast in X-Ray imagery, contrast enhancement is used as pre-processing step [47], [48]. Lung segmentation of chest X-Rays is a crucial and important step in order to identify lung nodules and various segmentation approaches are proposed in the literature [49]–[52].

**FIGURE 4.**
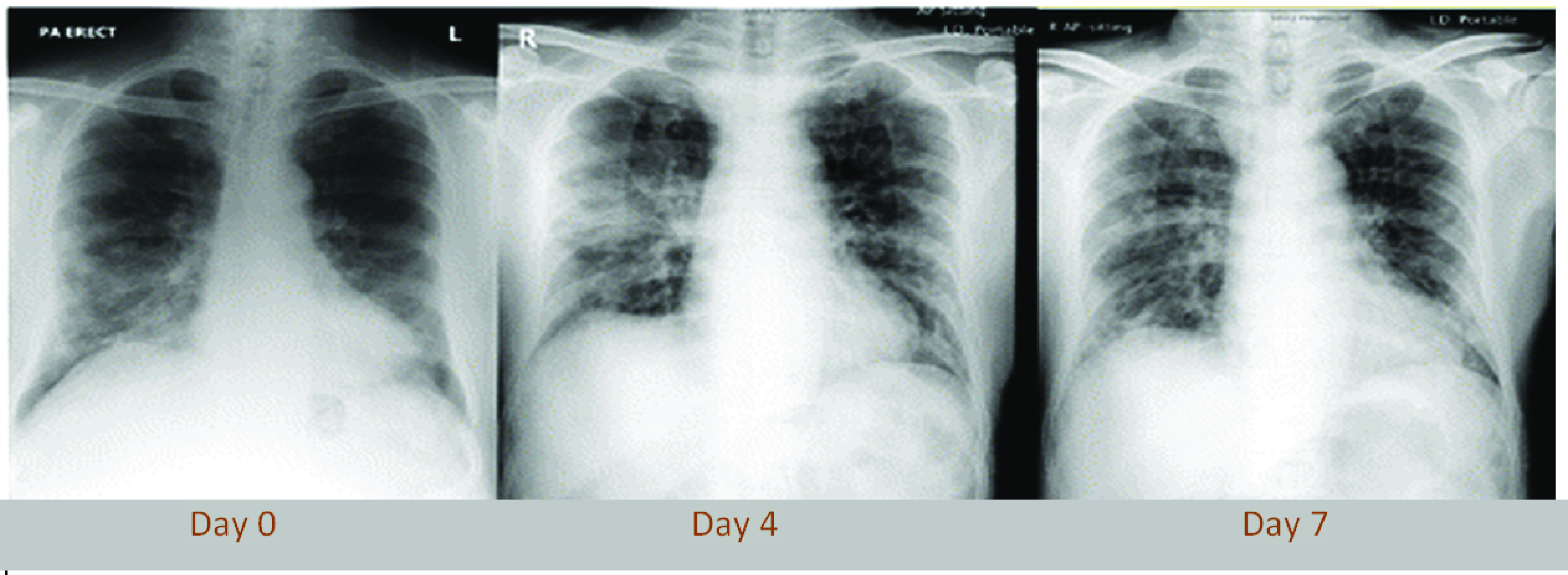
Chest CXR of an elderly male patient (Wuhan, China, who travelled to Hong Kong, China). Provided are three chest XR chosen out of the daily chest CXR for this patient. The consolidation can be observed in the right lower zone on day 0 persist into day four, followed by novel consolidate changes in the right mid-zone periphery and perihelia region. Such type of mid-zone change improved on the day seven-film. Image adapted from [41].

CXR examinations show consolidation in COVID-19 infected patients. In one study at Hong Kong [41], three different patients had daily CXR, two of them showed progression in the lung consolidation over 3–4 days. Further CXR examinations show improvement over the subsequent two days. The third patient showed no significant variations over eight days. However, a similar study showed that the ground glass opacities in the right lower lobe periphery on the CT are not visible on the chest radiograph, which was taken 1 hour apart from the first study. FIGURE 4 illustrates a scenario with three chest XR chosen out of the daily chest CXR for a patient. The consolidation can be observed in the CSR image. In a large-scale study of 636 ambulatory COVID-19 patients, Weinstock *et al.* found that 58% of CXR was normal and 89% were normal or mildly abnormal [53]. Interstitial changes (24%) and GGOs (19%) were the most prominent symptoms, and abnormalities were most prevalent in the lower lobe (34%). While the sensitivity of CXR is significantly lower than for CT, the American College of Radiology (ACR) recommends to conduct CXR with portable devices and only if “medically necessary” for better radiological analysis. It moreover firmly advises to not use any imaging technique for COVID-19 diagnosis but instead suggests biomolecular tests [54]. In the realm of AI,

Various CXR-related automated approaches are proposed. The following section discusses the most salient work, while Table 2 presents a more systematic presentation of such methods.

**TABLE 2:** Representative work for X-Ray based COVID-19 diagnosis

Study	Model	Dataset	Performance
Guszt’av Ga’al et al. [53]	Attention U-Net+ adversarial+ Contrast Limited Adaptive Histogram Equalization (CLAHE) [75]	247 images from Japanese Society of Radiological Technology (JSRT) Dataset+ Shenzhen dataset contains a total of 662 chest X-Rays	DSC of 97.5% on the JSRT dataset
Asmaa Abbas et al. [64]	CNN features of pre-trained models on ImageNet and ResNet+ Decompose, Transfer, and Compose (DeTraC), for the classification of COVID-19 chest X-Ray images: The developed code is available at https://github.com/asmaa4may/DeTraCCOVId19	I80 samples of normal CXRs (with 4020 $\times$ 4892 pixels) from the Japanese Society of Radiological Technology (JSRT) + Cohen JP. COVID-19 image data collection. https://githubcom/ieee8023/covid-chestxray-dataset. 2020;.	High accuracy of 95.12% (with a sensitivity of 97.91%, a specificity of 91.87%, and a precision of 93.36%)
Ali Narin et al. [76]	Pre-trained ResNet50 model with transfer learning	The open source GitHub repository shared by Dr. Joseph Cohen+Chest X-Ray Images (Pneumonia) https://www.kaggle.com/paultimothymooney/chest-xray-pneumonia	Accuracy (97% accuracy for InceptionV3 and 87% accuracy for Inception-ResNetV2).
Linda Wang et al. [42]	COVID-Net: lightweight residual projection expansion- projection-extension (PEPX) design pattern, Model is available publicly for open access at https://github.com/lindawangg/COVID-Net.	COVIDx dataset: 16,756 chest radiography images across 13,645 patient cases from two open access data repositories	Accuracy 92.4% on COVIDx dataset
Ezz El-Din Hemdan et al. [60]	COVIDX-Net: based on seven different architectures of DCNNs; namely VGG19, DenseNet201, InceptionV3, ResNetV2, Inception ResNetV2, Xception, and MobileNetV2	COVID-19 cases provided by Dr. Joseph Cohen and Dr. Adrian Rosebrock [63]	F1-scores of 89% and 91% for normal and COVID-19, respectively
Khalid EL ASNAOUI et al. [77]	Fined tuned versions of (VGG16, VGG19, DenseNet201, Inception-ResNet-V2, Inception-V3, Resnet50, MobileNet-V2 and Xception).	5856 images (4273 pneumonia and 1583 normal).	Resnet50, MobileNet-V2 and Inception-Resnet-V2 show highly satisfactory performance with accuracy (more than 96%).
Prabira Kumar Sethy et al. [78]	Deep features from Resnet50 + SVM classification	Data available in the repository of GitHub, Kaggle and Open-i as per their validated X-Ray images.	Resnet50 plus SVM achieved accuracy, FPR, F1 score, MCC and Kappa are 95.38%,95.52%, 91.41% and 90.76% Respectively.
Ioannis D. Apostolopoulos1 et al. [79]	Various fine-tune dmodels: VGG19, MobileNet, Inception,Inception Resnet V2, Xception	1427 X-Ray images. 224 images with confirmed Covid-19, 700 images with confirmed common pneumonia, and 504 images of normal conditions are included	Accuracy with Xception was the highest, 95.57%, sensitivity of 8% and specificity of 99.99%.
Biraja Ghoshal et al. [58]	Dropweights based Bayesian Convolutional Neural Networks (BCNN)	68 Posterior-Anterior (PA) X-Ray images of lungs with COVID-19 cases from Dr. Joseph Cohen’s Github repository, augmented the dataset with Kaggle’s Chest X-Ray Images (Pneumonia) from healthy patients, a total of 5941 PA chest radiography images across 4 classes (Normal: 1583, Bacterial Pneumonia: 2786, non-COVID-19 Viral Pneumonia: 1504, and COVID-19: 68).	Accuracy of 89.82% with BCNN at dropweights rate=3%
Muhammad Farooq, Abdul Hafeez [59]	3-step technique to fine-tune a pre-trained ResNet-50 architecture to improve model performance	COVIDx dataset	Accuracy of 96.23% (on all the classes) on the COVIDx dataset
Yu-Huan Wu et al. [74]	3-class classifier (healthy, COVID-19, non-COVID-pneumonia with Res2Net backbone. Segmenation model wih VGG-16 backbone	COVID-CS dataset (144,167 images, 750 patients of which are 400 COVID-19 positive)	95% sensitivity and 93% specificity

#### 4) Representative Work, Evaluation and Discussion

To date, many deep learning-based computer vision models for X-Ray COVID-19 were proposed. One of the most significant development is the model COVID-Net [58] proposed by Darwin AI, Canada. In this work, human-driven principled network design prototyping is combined with machine-driven design exploration to produce a network architecture for the detection of COVID-19 cases from chest X-Ray. The first stage of the human-machine collaborative design strategy is based on residual architecture design principles. The dataset used to train and evaluate COVID-Net is referred to as COVIDx [58] and comprise a total of 16,756 chest radiography images across 13,645 patient cases. The proposed model achieved 92.4% accuracy 80% sensitivity for COVID-19 diagnosis.

**FIGURE 5.**
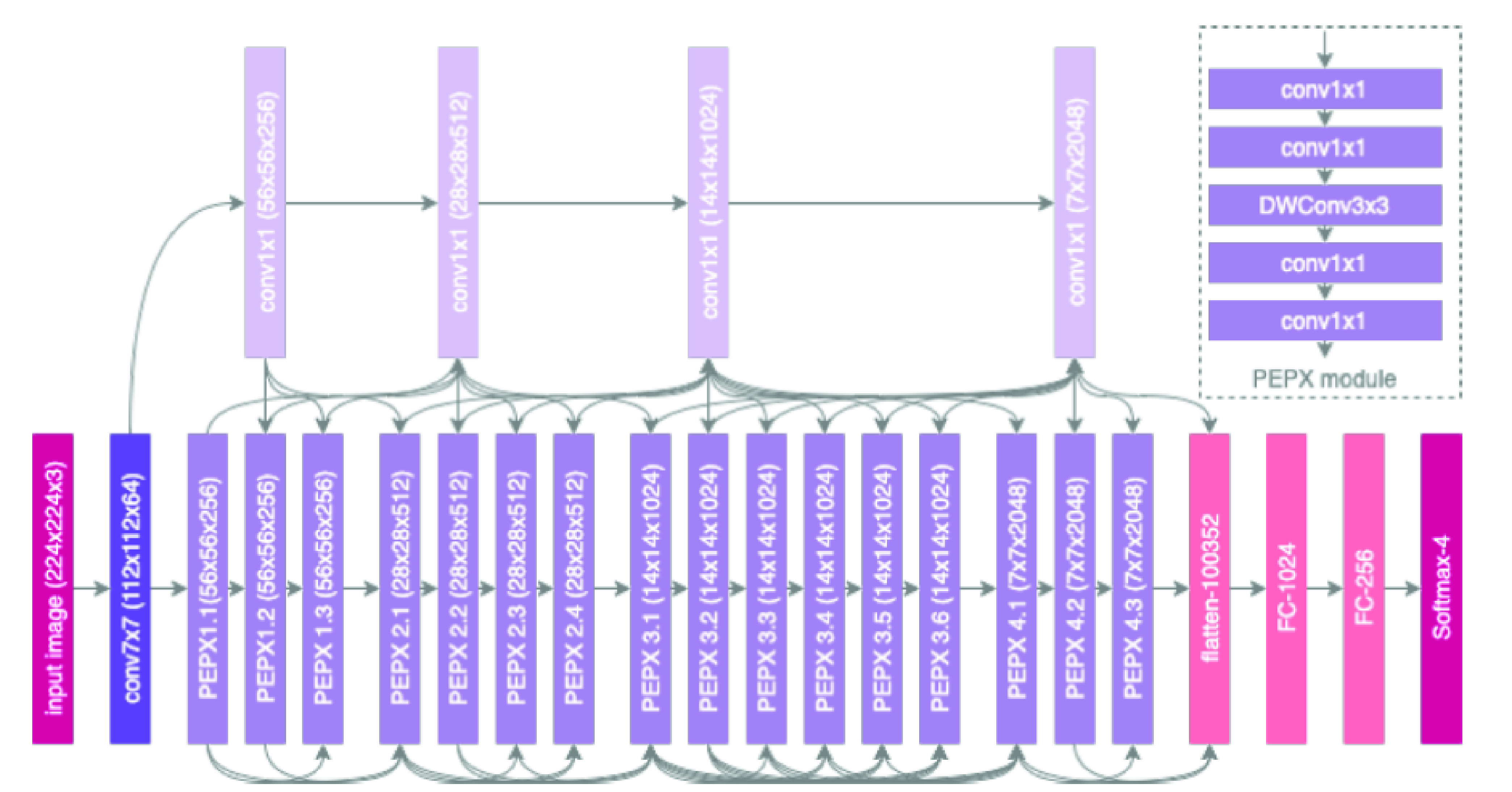
Architectural diagram of COVID-Net [42]. We can observe High architectural diversity and selective long-range connectivity.

The initial network design prototype makes one of three classes: a) no infection (normal), b) non-COVID19 infection (viral and bacterial), and c) COVID-19 viral infection. The goal is to aid clinicians to decide better which treatment strategy to employ depending on the cause of infection since COVID-19 and non-COVID19 infections require different treatment plans. In the second stage, data, along with human-specific design requirements, act as a guide to a design exploration strategy to learn and identify the optimal macro- and microarchitecture designs to construct the final tailor-made deep neural network architecture. The proposed COVIDNet network diagram is shown in FIGURE 5 and available publicly at https://github.com/lindawangg/COVID-Net.

Hemdan *et al.* [59] proposed the COVIDX-Net based on seven different architectures of DCNNs; namely VGG19, DenseNet201 [60], InceptionV3, ResNetV2, InceptionResNetV2, Xception, and MobileNetV2 [61]. These models were trained on COVID-19 cases provided by Dr Joseph Cohen and Dr Adrian Rosebrock, available at https://github.com/ieee8023/covid-chestxray-dataset [62]. The best model combination resulted in F1-scores of 0.89 and 0.91 for normal and COVID-19 cases. Similarly, Abbas *et al.* [63] proposed a Decompose, Transfer, and Compose (DeTraC) approach for the classification of COVID-19 chest X-Ray images. The authors applied CNN features of pre-trained models on ImageNet and ResNet to perform the diagnoses. The dataset consisted of 80 samples of normal CXRs (with 4020 $\times$ 4892 pixels) from the Japanese Society of Radiological Technology (JSRT) Cohen JP. COVID-19 image data collection, available at https://githubcom/ieee8023/covid-chestxray-dataset [62]. This model achieved an accuracy of 95.12% (with a sensitivity of 97.91%, a specificity of 91.87%, and a precision of 93.36%). The code is available at https://github.com/asmaa4may/DeTraCCOVId19.

Ghoshal and Tucker *et al.*. [57] introduced Uncertainty-Aware COVID-19 Classification and Referral model with the proposed Dropweights based on Bayesian Convolutional Neural Networks (BCNN). For COVID-19 detection to be meaningful, two types of predictive uncertainty in deep learning were used on a subsequent work [64]. One of it is Epistemic, or Model uncertainty accounts for the model parameters uncertainty as it does not take all of the aspects of the data into account or the lack of training data. The other is Aleatoric uncertainty that accounts for noise inherent in the observations due to class overlap, label noise, homoscedastic and heteroscedastic noise, which cannot be reduced even if more data were to be collected. Bayesian Active Learning by Disagreement (BALD) [65], is based on mutual information that maximizes the information between model posterior and predictions density functions approximated as the difference between the entropy of the predictive distribution and the mean entropy of predictions across samples.

A BCCN model was trained on 68 Posterior-Anterior (PA) X-Ray images of lungs with COVID-19 cases from Dr Joseph Cohen’s Github repository [62], augmented the dataset with Kaggle’s Chest X-Ray Images (Pneumonia) from healthy patients. It achieved 88.39% accuracy on the available dataset. This work additionally recommended visualisation of distinct features, as an additional insight to point prediction for a more informed decision-making process. It used the saliency maps produced by various state-of-the-art methods, e.g. Class Activation Map (CAM) [66], Guided Backpropagation, and Guided Gradient, and Gradients to show more distinct features in the CSR images.

A Capsule Network-based Framework called COVID-CAPS [67] is proposed for the Identification of COVID-19 cases from X-ray Images. A lightweight deep neural network (DNN) based mobile app is proposed in [68] that can process noisy images of chest X-ray (CXR) for point-of-care COVID-19 screening and is available at url:https://github.com/xinli0928/COVID-Xray. A 3-step approach to fine-tune a pre-trained ResNet-50 architecture to improve model performance is proposed by [58]. Similar other works are proposed recently [69]–[71].

To the best of our knowledge, [72] reported the largest dataset including 144,167 images from 750 patients (400 COVID patients). As deep-learning models are data-hungry and most other projects perform transfer learning on extremely small datasets (often < 1000 images), this is a remarkable project and a first step towards signifying more realistic and clinically relevant performance estimates. The classifier achieves a sensitivity of 95% and a specificity of 93%. Besides, a segmentation model is trained with the deep supervision strategy and shown to identify lesion areas of the positive predictions. One drawback of the work is that the models operate autonomously and the lesions identified by the segmentation model may by no means have been relevant for the positive prediction of the classifier.

#### 5) Ultrasound Imaging

Lung ultrasound (LUS) is evolved over the last few years to its theoretical and operative aspects. One of the characteristic features of LUS is its ability to define the alterations affecting the ratio between tissue and air in the superficial lung [55], [78].

The practical advantages of LUS are numerous: US devices are portable, bringing along the salient benefit of performing a point-of-care LUS at the patient’s bedside or even home that can easily be repeated for monitoring purposes. LUS minimizes the requirement for transferring the patient, controlling the potential risk of further infection and spreading it among health care personnel.

**FIGURE 6.**
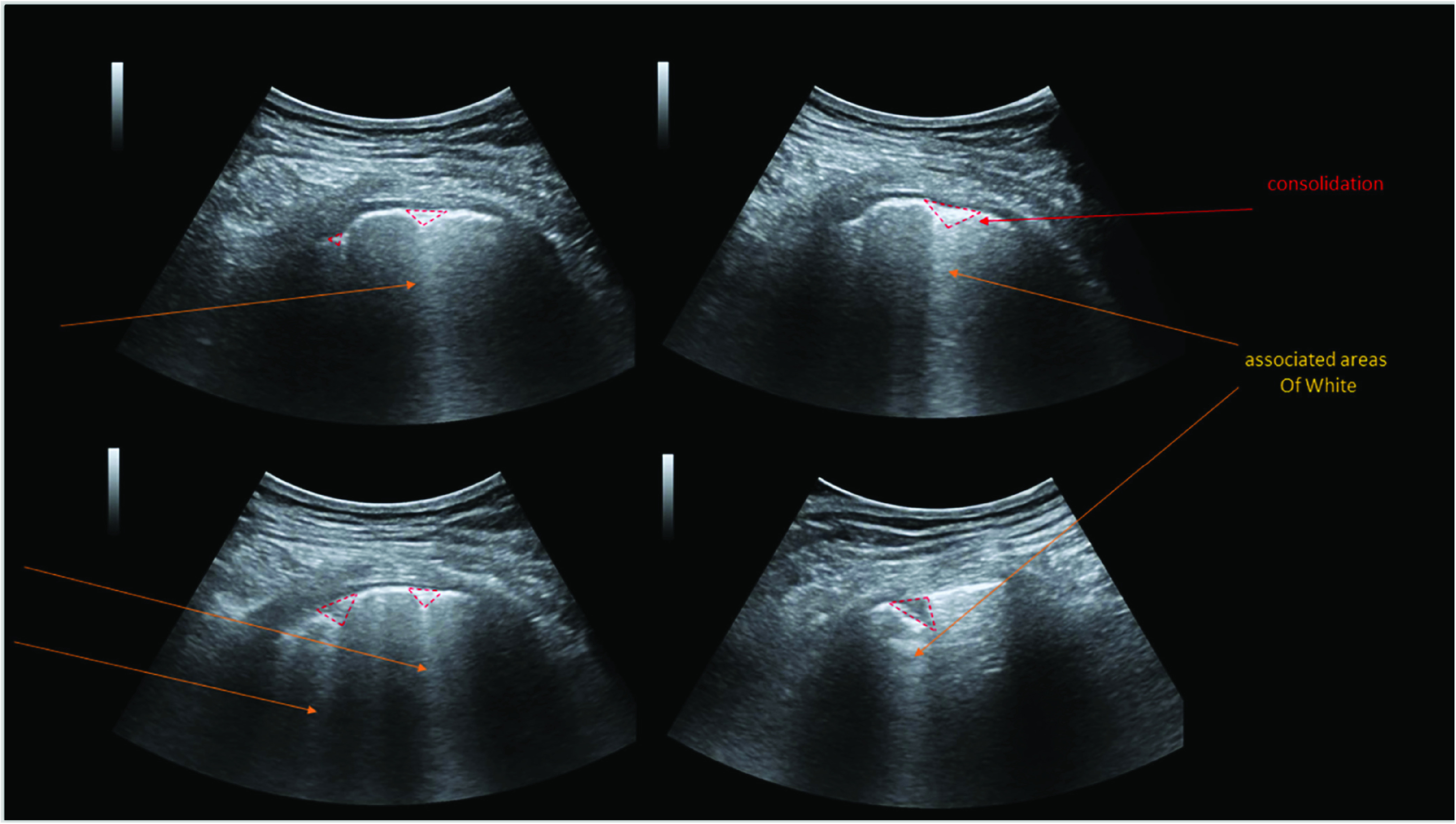
Detection of COVID-19 from ultrasound images: Ultrasound imagery is widely available and accessible throughout the world and therefore, can be a valuable tool for monitoring disease progression. Adapted from [55].

In contrast to CT and X-Ray, US is non-irradiating, and the instruments are cheap and thus highly available even outside developed countries [79]. However, ultrasound is operator-dependent and to follow standardized protocols for LUS like the BLUE protocol [80], experienced technicians are desired. This is boon and bane: While conducting a full LUS can take a few minutes and cause significantly higher portions of data than other modalities, the auto-correlation is exceptionally high and diagnostic patterns are visible only in few frames. LUS was repeatedly shown superiority to CXR for diagnosing pulmonary diseases (for review see [81]), especially in resource-limited settings [82]. For COVID-19, LUS patterns are correlated to disease stage, comorbidities and severity of pulmonary injury [83] and most dominantly include B-lines, vertical artifacts that range from the pleural deep into the lung [84]. Importantly, LUS was lately reported higher sensitivity and equal specificity than CXR in diagnosing COVID-19 [85]. In a comparison of LUS to CT, it was shown that for all typical features of LUS in COVID-19 patients, analogs to known patterns in CT scans could be found [86]. FIGURE 6 illustrates the detection of COVID-19 from ultrasound images. While LUS is used commonly as a first-line examination method in European countries like Italy [87], it is not mentioned in the ACR recommendations as clinical practice for COVID [54]. Besides, some articles argued that LUS can assist early diagnosis and assessment of COVID and even found better sensitivity of LUS in detecting certain features [88]. This has caused a vivid debate on the role of LUS for the COVID pandemic [89]–[92].

#### 6) Representative Work, Evaluation and Discussion

Since LUS is a less established practice for examining COVID-19 patients, less clinical data is recorded and publicly available. It is presumably a primary reason why fewer computer vision projects focus on it, despite the advocacy of recent trends in medicine (see above).. TABLE 3 presents a more categorical presentation of such methods.

**TABLE 3:** Representative Works for Infected Disease Prevention and Control

Study	Prevention Control Methodology	Implementation/Dataset	Performance
Zhongyuan Wang et al. [102]	Masked Face Recognition based on deep learning	Dataset is available at: https://github.com/X-zhangyang/Real-World-Masked-Face-Dataset.	The multigranularity masked face recognition model we developed achieves 95% accuracy
Joshua M. Pearce [103]	RepRap-class 3-D printers and open source microcontrollers, mass distributed manufacturing of ventilators		3D printing can facilitate supply chain.
W. Chiu, et al. [104]	Inrfrared thermograpgy: Mass-fever screening	72,327 patients or visitors passed through the only entrance where a thermography station was in operation.	Over a period of one month, hundred and five patients or visitors were detected to have a thermographic fever detection
Edouard A. Hay [105]	Convolutional neural networks for identification of bacteria: Github repository https://github.com/rplab/Bacterial-Identification.	Light sheet microscopy image data	Over 90% accuracy

Preliminary investigations for clarifying the diagnostic and prognostic role of LUS in COVID-19 are underway. Computer vision on ultrasound imaging became increasingly popular in the last years [93], but comparably little work has been done on LUS.

The first work to apply computer vision on ultrasound probes of COVID-19 patients was POCOVID-Net, a deep convolutional neural network with a VGG backbone [94]. POCOVID-Net introduced an LUS dataset that initially consisted of 1103 images (654 COVID-19, 277 bacterial pneumonia, and 172 healthy controls), sampled from 64 videos. As of July 2020, the dataset contains ~150 videos and ~50 images, resembling the largest publicly available dataset of LUS: https://github.com/jannisborn/covid19_pocus_ultrasound. Besides, the trained models were deployed and can be freely used at: https://pocovidscreen.org. On the initial dataset, POCOVID-Net reports a video accuracy of 92% and a sensitivity and specificity of 96% and 79% for COVID-19 respectively. It accounts for a preliminary proof-of-concept that COVID-19 can be automatically distinguished from other pulmonary conditions through LUS, and it opens a branch to follow up on the granularity of the differentiation.

On the updated POCOVID-Net dataset, performance could be improved with an accuracy of 94%, sensitivity and specificity of 98% and 91% in a 5-fold cross-validation on LUS videos [95]. This work utilizes Bayesian deep learning to compute uncertainty estimations that are deemed crucial for medical imaging [96]. [95] then demonstrated how epistemic uncertainty estimations (measured by Monte Carlo dropout) could let the model self-recognize low confidence situations. Additionally, the authors computed and validated CAMs with the help of medical experts and found that the model learns in a completely unsupervised fashion to highlight lung consolidations (94% sensitivity) and, to a lesser extent, A-lines (62%).

The CAMs were overall found helpful for diagnosis by the experts. However, it leaves room for improvement in B-line detection. Interestingly, the performance could be mildly improved when the classifier was coupled with the segmentation model by [97].

The named work by [97] introduced a rich stack of CNN models for segmentation and severity assessment of COVID-19 patients. Based on ~1000 images from convex probes of 33 patients, an ensemble of 3 segmentation models (UNet, UNet++ and Deeplabv3+) is shown to reliable extract both, A-lines and COVID biomarkers (accuracy 96%, binary dice score 0.75). Besides, they classify COVID severity on four levels (0 to 3). They introduce a so-called regularised spatial transformer network that performs a weak localization by extracting two transformed image sections that, ideally, should contain pathological artifacts. Their model achieves a precision of 70% and a recall of 60% on the four-class classification. However, despite the authors claim to release a dataset of 277 LUS videos from 35 patients with a total of almost 60,000 frames, to date, only 60 videos can be accessed (after the account request is manually approval). No annotations are available for those videos, rendering a validation of the results effectively impossible.

As B-lines are maybe the most critical LUS feature in COVID patients, [98] presented a specialized approach for line artifact quantification that utilizes a non-convex regularization technique dubbed Cauchy proximal splitting. This technique outperforms state-of-the-art B-line identification [99] and detects 87% of the B-lines in 9 COVID-19 patients, reducing the error margin by 40% compared to [99].

Since ultrasound equipment is small and portable options are available (POCUS devices), the impact of web-independent, on-device analysis is high, especially since LUS belongs to the standard repertoire even in remote medical facilities.

Future projects could, for example, improve the mediocre results found in an ablation study with mobile-friendly CNNs [95] to facilitate on-device processing.

### B. Prevention and Control

WHO has provided some guidelines on infection prevention and control (IPC) strategies for use when infection with a novel coronavirus is suspected [104]. Major IPC tries to control transmission in health care settings that include early recognition and source control and applying standard precautions for all patients. It also includes implementation of additional empiric precautions like airborne precautions for suspected cases of COVID-19, implementation of administrative controls, and use of environmental and engineering controls. Computer vision applications are providing valuable support for the implementation of IPC strategies.

#### 1) Representative Work, Evaluation and Discussion

Protective techniques to control the virus spread in the early stage of disease progression were considered very early, as the usage of masks. Some countries like China implemented it as a control strategy at the start of the epidemic. Computer vision-based systems greatly facilitated such implementation.

Wang *et al.*. [100] proposed the Masked Face Recognition approach using a multi granularity masked face recognition model, resulting in 95% accuracy on a masked face image dataset. The data was made public for research and provided three types of masked face datasets, including Masked Face Detection Dataset (MFDD), [105], Real-world Masked Face Recognition Dataset (RMFRD) and Simulated Masked Face Recognition Dataset (SMFRD) [106].

**FIGURE 7.**
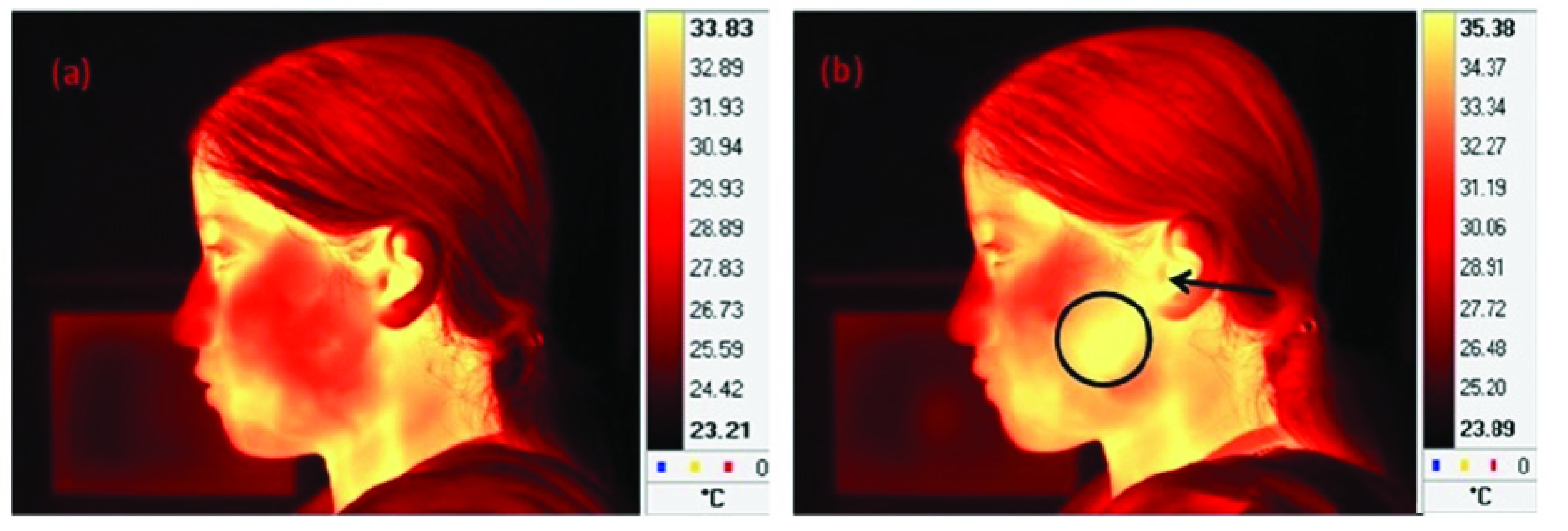
Temperature screening in process with thermal imagery of a subject who is talking on a mobile phone; (a) after 1 min of talking and (b) after 15 min of talking. It shows that the temperature of the encircled region increased from 30.56 to 35.15 C after 15 min of talking. The temperature of the region around the ear (indicated by an arrow) elevated from 33.35 to 34.82C. A similar system can be used for COVID-19 related fever screening.Adapted from [56].

A similar strategy is the use of Infrared thermography. It can be used as an early detection strategy for infected people, especially in crowns like passengers at an airport-various medical applications of infrared thermography re summarised by Lahiri *et al.* [56], including fever screening. Somboonkaew *et al.* [107] introduced a mobile platform that can be used for an automatic fever screening system using forehead temperature. Ghassemi *et al.* [108] has discussed the best practices for standardized performance and testing of infrared thermographs. An Infection Screening System based on Thermography and CCD Camera is proposed by Negishi *et al.* [109] with Good Stability and Swiftness for Non-contact Vital-Signs Measurement by Feature Matching and MUSIC Algorithm. Earlier for SARD spread control. A computer vision system to help in fever screening by Chiu *et al.* [102] was used in earlier outbreaks of SARS. From 13 April to 12 May 2003, 72,327 patients and visitors passed through the only entrance allowed at TMU-WFH where a thermography station was in operation. FIGURE 7 illustrates the use of thermal imagery for temperature screening.

Additional miscellaneous approaches for prevention and control are also worth noting. An example is pandemic drones using remote sensing and digital imagery, which were recommended for identifying infected people. Al-Naji *et al.* [110] have used such a system for remote life sign monitoring in disaster management in the past. A similar application is to use vision-guided robot control for 3D object recognition and manipulation. Moreover, 3D modelling and printers are helping to maintain the supply of healthcare equipment in this troubled time. Pearce [101] discusses RepRap-class 3-D printers and open-source microcontrollers. The applications are relevant since mass distributed manufacturing of ventilators has the potential to overcome medical supply shortages. Lastly, germ scanning is an essential step against combating COVID-19. Hay and Parthasarathy [103] has proposed a convolutional neural network for germ scanning such as the identification of bacteria Light-sheet microscopy image data with more than 90% accuracy.

### C. Treatment and Clinical Management

**FIGURE 8.**
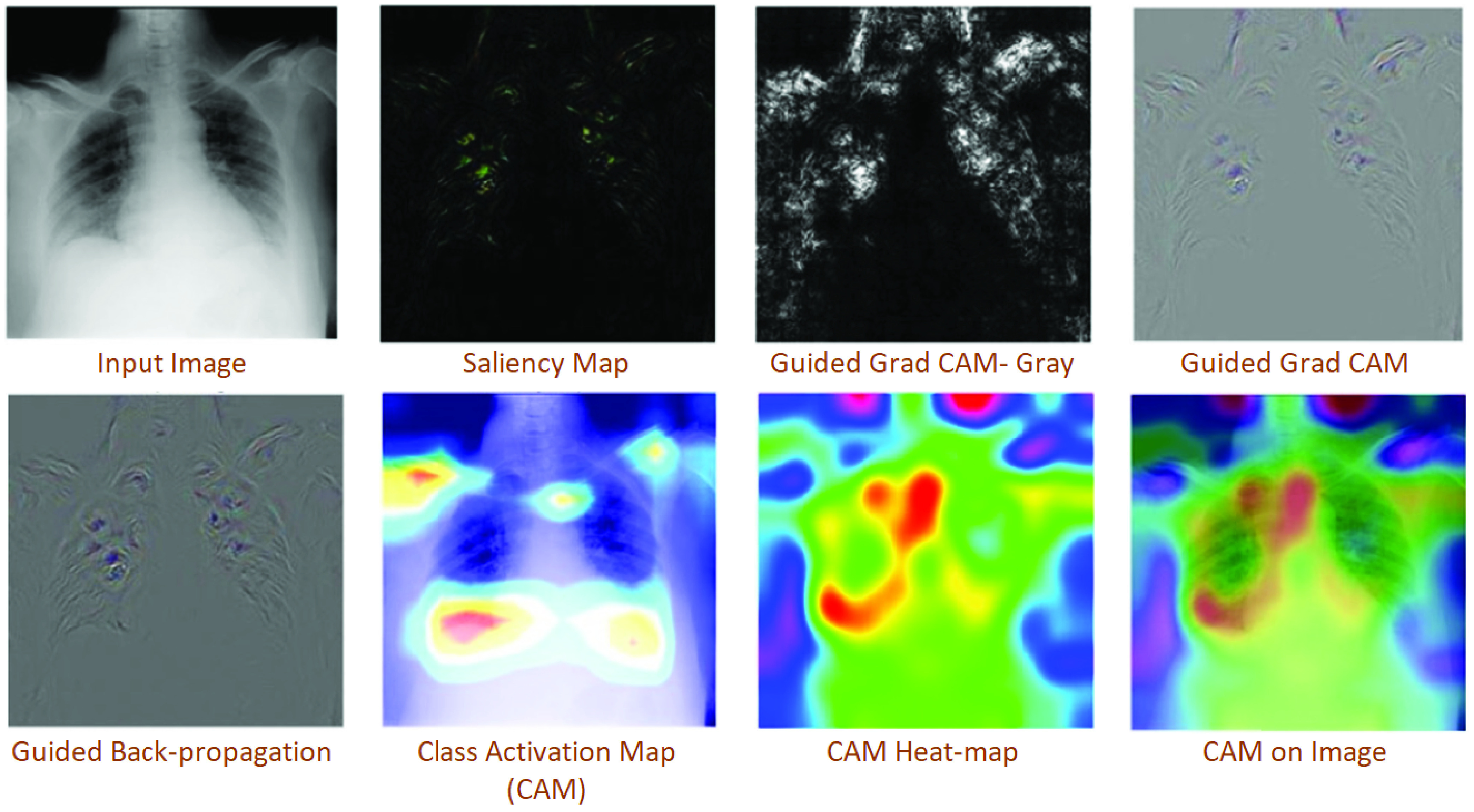
Visualizations shown by using different saliency maps that provide additional insights diagnosis. These maps help to identify the areas of activation that can lead to disease progression monitoring and severity detection. Adapted from [57].

**FIGURE 9.**
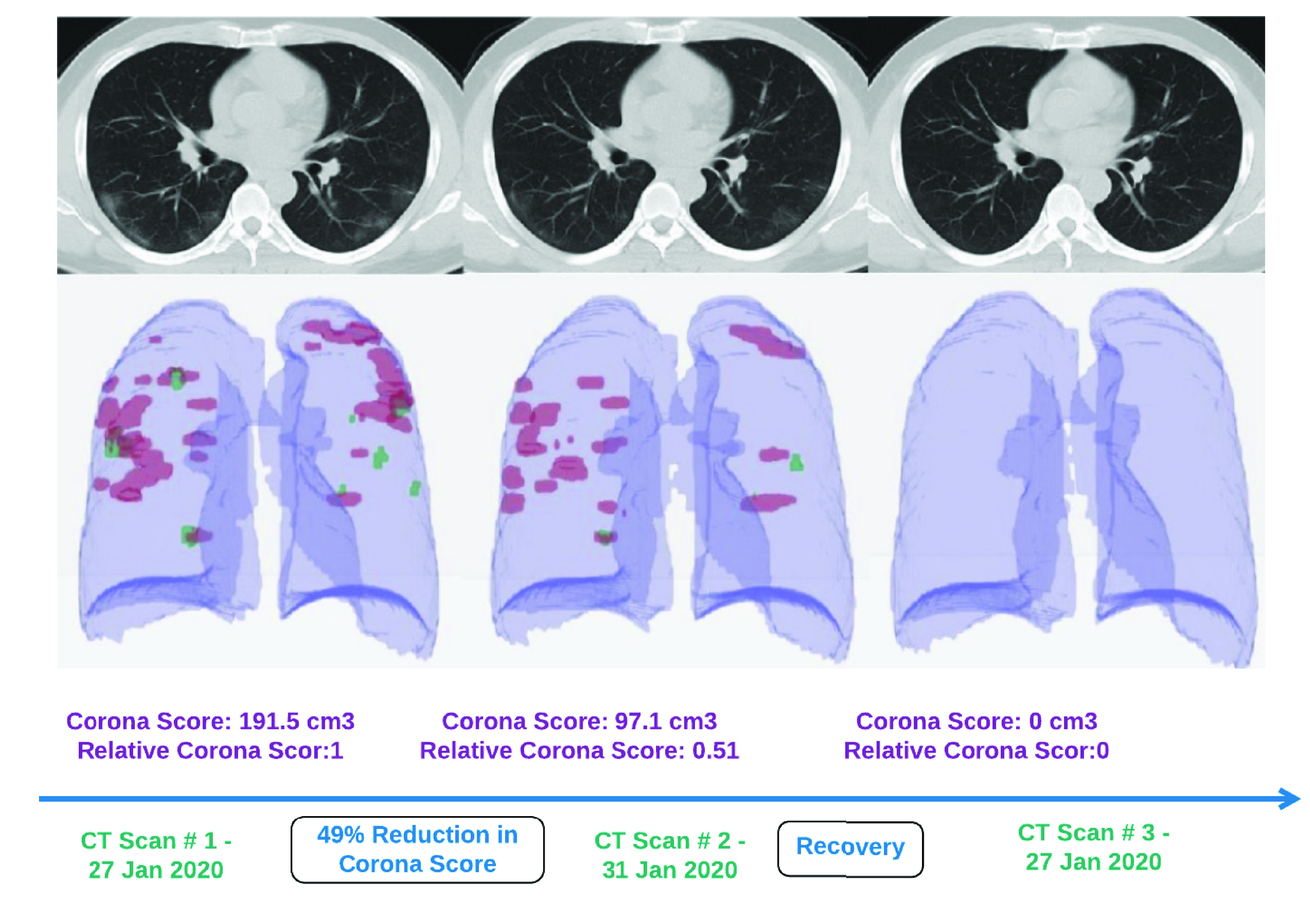
Method of corona score calculation for patient disease progression monitoring is illustrated. It is one of the ways infected areas can be visualised, and disease severity can be predicted for better disease management and patient care. [35].

Although various attempts and claims of vaccinations development are announced in the media, however, there is no agreed and widely used treatment for disease caused by the virus at the moment. However, many of the COVID-19 symptoms can be treated.depending on the clinical condition of the patient. An improvement in clinical management practices is possible through automating various practices with the help of computer vision. One example is the classification of patients based on the severity of the disease and advising them appropriate medical care. FIGURE 8. provides a scenario of progression and severity monitoring by using different saliency maps that provide additional insights diagnosis. These maps help to identify the areas of activation that can lead to disease progression monitoring and severity detection. FIGURE 9 illustrates the Corona score calculation on a 3D model of patients CT images for patient disease progression. It is one of the ways infected areas can be visualised, and disease severity can be predicted for better disease management and patient care. TABLE 4 presents a more categorical presentation of such methods.

**TABLE 4:** Representative Works for Infected Disease Treatment and Progression Monitoring

Study	Treatment Or Management Methodology	Implications
Daniel Wrapp et al. [115]	Using biophysical assays, it is shown that this protein binds at least 10 times more tightly than the corresponding spike protein of severe acute respiratory syndrome (SARS)-CoV to their common host cell receptor.	The virus connects to host cells through its trimeric spike glycoprotein. 3.5-angstrom-resolution cryo-electron microscopy structure of the 2019-nCoVS trimer in the prefusion conformation was studied These studies provide valuable. information to guide the development of medical counter-measures for 2019-nCoV.
Ophir Gozes et al. [35]	Corona score for patient disease progression monitoring and screening	It was basedonthe development of CT images Dataset provide d by ChainZ (www.ChainZ.cn) and Corona score was used to screen critically ill patients. For instance, Corona score of 191.5 cm3 was observed at the time of Admission and after recovery, it turned 0 means no opacities
Yunlu Wang et. al. [114]	Depth camera and deep learning is used to classify 6 clinically significant respiratory patterns. GRU neural network with bidirectional and attentional mechanisms (BI-AT-GRU)	Abnormal respiratory patterns classifier can be able to large-scale screening of people infected with COVID-19
Yoshihiro Uesawa et. al. [118]	Quantitative structure-activity relationship analysis with the help of deep learning	Potential use in Drug discovery

#### 1) Representative Work, Evaluation and Discussion

An essential part of the fight against the virus is clinical management, which can be done by identifying patients that are critically ill so that they get immediate medical attention or ventilator support. A disease progression score is recommended to classify different types of infected patients in [35]. It is called “corona score” and is calculated by measurements of infected areas and the severity of disease from CT images. The corona score measures the progression of patients over time, and it is computed by a volumetric summation of the network-activation maps.

MacLaren *et al.* [111] supports that radiological evidence can also be an essential tool to distinguish critically ill patients. Wang *et al.* [112] used depth camera and deep learning as abnormal respiratory patterns classifier that may contribute to the large-scale screening of people infected with the virus accurately and unobtrusively. Respiratory Simulation Model (RSM) is developed to control the gap between scarce real-world data and a large amount of training data. They proposed GRU neural network with bidirectional and attentional mechanisms (BI-AT-GRU) to classify six clinically significant respiratory patterns (Eupnea, Tachypnea, Bradypnea, Biots, Cheyne-Stokes, and Central-Apnea) to identify critically ill patients. The proposed model can classify the respiratory patterns with accuracy, precision, recall, and F1 of 94.5%, 94.4%, 95.1%, and 94.8%, respectively. Demo videos of this method working in situations of one subject and two subjects can be accessed online (https://doi.org/10.6084/m9.figshare. 11493666.v1).

The CoV spike (S) glycoprotein is the main target for vaccines, therapeutic antibodies, and diagnostics that can guide future decisions. The virus connects to host cells through its trimeric spike glycoprotein. Using biophysical assays, Wrapp *et al.* [113] illustrated that this protein binds to their common host cell receptor at least ten times more tightly than the corresponding spike protein of severe acute respiratory syndrome (SARS)-CoV. Protein X-ray crystallography can discover the atomic structure of molecules and their functions. It can further facilitate scientists to design new drugs targeted to that function. MAchine Recognition of Crystallization Outcomes (MARCO) [114] initiative has introduced deep convolutional networks to achieve an accuracy of more than 94% on the visual recognition task of identifying protein crystals. It uncovers the potential of computer vision and deep learning for drug discovery.

Quantitative structure-activity relationship (QSAR) analysis has perspectives on drug discovery and toxicology [115]. It employs structural, quantum chemical and physicochemical features calculated from molecular geometry as explanatory variables predicting physiological activity. Deep feature representation learning can be used for QSAR analysis by incorporating 360° images of molecular conformations. Uesawa [116] has proposed QSAR (Quantitative structure-activity relationship) analysis using deep learning using a novel molecular image input technique. Similar techniques can be used for drug discovery to pave the way for vaccine development for COVID-19.

## IV. Dataset and Resources

### A. CT Images

• COVID-CT-Dataset [117] - The University of San Diego has released a data set with 349 CT images containing clinical findings of COVID-19. It claims to be the largest of its kind. To demonstrate its potential, an AI model is trained, achieving 85% accuracy. The data set is available at https://github.com/UCSD-AI4H/COVID-CT.
• An image-based model working with CTs for COVID-19 diagnosis can be found at https://github.com/JordanMicahBennett/SMART-CT-SCAN_BASEDCOVID19_VIRUS_DETECTOR/.

### B. CX-Ray Images

• COVID-19 Radiography database [118] - A team of researchers from Qatar University, Doha, and the University of Dhaka, Bangladesh, along with collaborators from Pakistan and Malaysia with medical doctors have created a database of chest X-Ray images for COVID-19 positive cases along with Normal and Viral Pneumonia images. In the current release, there are 219 COVID-19 positive images, 1341 normal images and 1345 viral pneumonia images. The authors said that they would continue to update this database as soon as new X-Ray images for COVID-19 pneumonia patients. The project can be found at GitHub with MATLAB codes and trained models: https://github.com/tawsifur/COVID-19-Chest-X-Ray-Detection. The research team managed to classify COVID-19, Viral pneumonia and Normal Chest X-Ray images with an accuracy of 98.3%. This scholarly work was submitted to Scientific Reports (Nature), and the manuscript was uploaded to ArXiv. Please make sure to give credit while using the dataset, code and trained models.
• COVID-19 Image Data Collection [62]- An initial COVID-19 open image data collection is provided by Joseph Paul Cohen. all images and data are released under the following URL https://github.com/ieee8023/covid-chestxray-dataset.
• COVIDx Dataset [42] - This is the release of the brand-new COVIDx dataset with 16,756 chest radiography images across 13,645 patient cases. The current COVIDx dataset is constructed by the open-source chest radiography datasets at https://github.com/ieee8023/covid-chestxray-dataset and https://www.kaggle.com/c/rsna-pneumonia-detection-challenge. It is a combination of data provided by many parties: the Radiological Society of North America (RSNA), others involved in the RSNA Pneumonia Detection Challenge, Dr Joseph Paul Cohen, and the team at MILA, involved in the COVID-19 image data collection project for making data available to the global community.
• Chest X-Ray8 [119] - The chest X-Ray is one of the most commonly accessible radiological examinations for screening and diagnosis of many lung diseases. A tremendous number of X-Ray imaging studies accompanied by radiological reports are accumulated and stored in many modern hospitals’ Picture Archiving and Communication Systems (PACS), available at https://nihcc.app.box.com/v/ChestXray-NIHCC).

### C. Other Images

• Lung ultrasound dataset [94] - An open data collection initiative similar of LUS, similar to the one by Cohen *et al.* for CT and CXR. The growing database is continuously updated and while it partially collects data from dispersed public sources it also releases unpublished clinical data. The dataset is thought to facilitate differential diagnosis from LUS and provides 4 classes (healthy, bacterial pneumonia, COVID-19 and non-COVID viral pneumonia. As of July 2020, the dataset contains ~150 videos and ~50 images resembling the largest publicly available dataset of LUS: https://github.com/jannisborn/covid19_pocus_ultrasound.
• Masked Face Recognition Datasets [100] - Three types of masked face datasets were introduced, including Masked Face Detection Dataset (MFDD), Real-world Masked Face Recognition Dataset (RMFRD) and Simulated Masked Face Recognition Dataset (SMFRD). MFDD dataset can be used to train an accurate masked face detection model, which serves for the subsequent masked face recognition task. RMFRD dataset includes 5,000 pictures of 525 people wearing masks and 90,000 images of the same 525 subjects without masks. To the best of our knowledge, this is currently the world’s largest real-world masked face dataset. SMFRD is a simulated masked face data set covering 500,000 face images of 10,000 subjects. These datasets are available at https://github.com/X-zhangyang/Real-World-Masked-Face-Dataset.
• Thermal Images Datasets - There is no dataset of thermals for high fever screening. However, a fully annotated thermal face database and its application for thermal facial expression recognition were proposed by Kopaczka [120]. Information on further ideas of related data that can be figured out by using such systems is available at http://www.flir.com.au/discover/public-safety/thermal-imaging-for-detecting-elevated-body-temperature/.

## V. Discussion and Future Work

Overall, it is encouraging that the computer vision research community had a massive response in return to the call for fighting COVID-19 epidemic. Data was collected and shared in a short time, and researchers proposed various approaches to address different challenges related to disease control. It became possible due to recent success in the field of deep learning and artificial intelligence. Web repositories like GitHub and ArXiv have contributed significantly to the rapid sharing of information. However, the impact of this research work is limited due to lack of clinical testing, fair evaluation and appropriate imaging datasets.

We argue that COVID-19 research landscape is quite broad that covers more than imaging and becomes beyond the scope of computer vision research. Similarly, we did not include any machine learning or signal processing work that does not include imaging modality. Most of the research work is performed around disease diagnosis problem with various performance metrics and without clinical trials that make it hard to compare their performance.

Similarly, various research datasets have been released for research purpose since the outbreak of the epidemic. However, these datasets can offer only limited scope and problem domains. For instance, for disease progression, often multiple images related to single patients are required with the timeline. Similarly, to evaluate different imaging modalities, researchers require multimodal imaging data related to the same patient that is not yet available for research purposes. The future work includes the fair performance comparison of different approaches, collection of a vast universal dataset and benchmark. We hope that the collective efforts of computer vision community like Imagaenet challenge can fill up this gap.

## VI. Concluding Remarks

In this article, we presented an extensive survey of computer vision efforts and methods to combat the COVID-19 pandemic challenge and also gave a brief review of the representative work to date. We divide the described methods into four categories based on their role in disease control: Computed Tomography (CT) scans, X-Ray Imagery, Ultrasound imaging and Prevention and Control. We provide detailed summaries of preliminary representative work, including available resources to facilitate further research and development. We hope that, in this first survey on Computer vision methods for COVID-19 control with extensive bibliography content, one can find give valuable insight into this domain and encourage new research. However, this work can be considered only as an early review since many computer vision approaches are being proposed and tested to control the COVID-19 pandemic at the current time. We believe that such efforts will be having a far-reaching impact with positive results to periods during the outbreak and post the COVID-19 pandemic.
